# A novel hazard assessment method for biomass gasification stations based on extended set pair analysis

**DOI:** 10.1371/journal.pone.0185006

**Published:** 2017-09-22

**Authors:** Fang Yan, Kaili Xu, Deshun Li, Zhikai Cui

**Affiliations:** 1 School of Resources and Civil engineering, Northeastern University, Shenyang, P. R. China; 2 School of Environmental and Chemical Engineering, Shenyang Ligong University, Shenyang, P. R. China; 3 Jacobs schools of engineering, University of California, San Diego, CA, United States of America; Leibniz-Institut fur Pflanzengenetik und Kulturpflanzenforschung Gatersleben, GERMANY

## Abstract

Biomass gasification stations are facing many hazard factors, therefore, it is necessary to make hazard assessment for them. In this study, a novel hazard assessment method called extended set pair analysis (ESPA) is proposed based on set pair analysis (SPA). However, the calculation of the connection degree (CD) requires the classification of hazard grades and their corresponding thresholds using SPA for the hazard assessment. In regard to the hazard assessment using ESPA, a novel calculation algorithm of the CD is worked out when hazard grades and their corresponding thresholds are unknown. Then the CD can be converted into Euclidean distance (ED) by a simple and concise calculation, and the hazard of each sample will be ranked based on the value of ED. In this paper, six biomass gasification stations are introduced to make hazard assessment using ESPA and general set pair analysis (GSPA), respectively. By the comparison of hazard assessment results obtained from ESPA and GSPA, the availability and validity of ESPA can be proved in the hazard assessment for biomass gasification stations. Meanwhile, the reasonability of ESPA is also justified by the sensitivity analysis of hazard assessment results obtained by ESPA and GSPA.

## 1. Introduction

Renewable energy has played an important role in the energy consumption all over the world. As an important part of renewable energy, the development of biomass energy gives a positive impact on economic growth [1]. Moreover, biomass energy is one of the critical solutions for future energy shortages [2], and it has met a rapid development worldwide recently [3–6]. Multifarious kinds of technologies are involved in biomass energy, including methane production [7], biodiesel [8], biomass to liquid (BTL) [9], biomass gasification [10], *etc*. However, biomass gasification technology has developed drastically due to the growing attention of the renewable and sustainable energy [10]. In China, a mass of biomass gasification stations have been put into service in rural areas. These stations can be utilized to produce biomass energy, *i*.*e*., the biomass gas. What's more, the burning of crop straw which leads to air pollution can be reduced by the them [11]. However, hazard factors existed in the process of biomass gasification will lead to fire, explosion, and poisoning accidents [12–14]. Cummer and brown [12] indicated that the biomass gas is poisonous, and people can be poisoned by the leakage of biomass gas. With regard to the study of Molino *et al*. [14], they emphasized that fire and explosion risks exist in the biomass gasification plant. Owing to the development of biomass gasification stations will be limited by the frequent occurrence of these accidents, hazard assessment is needed to evaluate hazard factors in the biomass gasification station [15,16].

As general terms of methods for the evaluation of hazards, hazard assessment is practical and effective in the safety management for accident hazards. Kinds of methods are involved in the hazard assessment which is also called the risk assessment, including risk assessment methods based on indices [17,18], fuzzy methods [19], analytic hierarchy process (AHP) [20], set pair analysis (SPA) [21], *etc*. In particular, SPA is a practical method which was proposed by a Chinese scholar to make a comprehensive analysis of certain and uncertain information [22]. As an improved uncertainty theory [23], SPA considers both certainties and uncertainties for a given system, and certainties and uncertainties are depicted from three aspects as identity, discrepancy, and contradistinction [24]. The evaluation of biomass gasification stations is hard to make due to the complication of hazard factors. Therefore, SPA can be an effective method in the hazard assessment for biomass gasification stations. In retrospect, SPA has met many improvements and developments so that it can be used in various areas. Yang *et al*. [25] proposed a nonlinear optimization set pair analysis model (NOSPAM) to evaluate the water resource renewability of the Yellow River Basin, in their works, subjective and objective information were optimized so that the weight can be confirmed based on the gray-encoded hybrid accelerating genetic algorithm. The assessment results showed that NOSPAM can not only make calculation of the weight, but also make quantification of the uncertain information in the water resource renewability assessment. In the study of Jin *et al*. [26], SPA was coupled with BP neural network to establish a new forewarning model called BPSPA-FM, and the back-propagation neural network updating model was introduced to confirm evaluation index values. According to the forewarning results for sustainable utilization of regional water resources, the BPSPA-FM was proved to be reasonable in the application of early warning for different natural hazards. Wang *et al*. [27] made modification of constant weights in the SPA, and dynamic weights were proposed by them, then they made a feasible and effective evaluation of the liquefaction so that evaluated samples can be described quantitatively. Jiang *et al*. [28] combined SPA with Quadrant Method to make comprehensive assessment of river ecosystem, two-dimensional Quadrant Method was introduced in their study to reveal the internal logic relation of the river ecosystem's structure and function, thereby determining the assessment zoning in SPA. Fuzzy methods were employed into SPA to determine the effects of land consolidation on the multifunctionality of the cropland production system in the study of Guo *et al*. [29], they took advantage of variable fuzzy sets analysis (VFSA) to quantify the influence of land consolidation, and then the quantitative data can be used in SPA. In the prediction analysis of integrated carrying capacity (ICC) using SPA, Wei *et al*. [30] introduced the theory of Euclidean geometry and the nearest recognition principle to reflect the effectiveness of prediction results by SPA. Yu *et al*. [31] proposed the improved five-element connectivity degree for the SPA, then they used the improved method to make a comprehensive evaluation of the groundwater quality. In contrast with fuzzy comprehensive evaluation results, the validity of the improved method can proved for the detection of the polluted water. Wang and Zhou [32] developed a coordinated development model based on the SPA. With regard to their method, the Identical-Discrepancy-Contrary (IDC) ranking system was proposed to assess the coordination ability, and assessment results can provide information for the sustainable social-ecological systems development. Chong *et al*. [33] utilized the SPA to assess the occupational hazard of coal mining. A novel assessment model was proposed by them using the SPA, Delphi method and AHP. Research results showed that the proposed model can describe the dynamic process and give a new resolution for the decision-making of uncertain and complicated environment. In addition, SPA also can be effectively utilized in the risk assessment of the enterprise management [34], flood disaster [21,35] and major hazard installations [24], hazard degree assessment of landslide [36], dam leakage investigation [23], disease diagnosis [37], risk assessment of water pollution sources [38], safety assessment of thermal power plants [39], statistical prediction of water resources [40], hazard assessment of debris flow [41], *etc*.

As previously mentioned, it can be concluded that SPA is a valid method to make hazard or risk assessment in various fields, including flood [21,35], coal mining [33], enterprise management [34], landslide [36], water pollution sources [38], thermal power plants [39], and debris flow [41]. However, the hazard assessment for biomass gasification stations using SPA is rare. It can be sought that biomass gasification stations have just been made hazard assessment by Yan *et al*. [42] using general set pair analysis (GSPA). In their study, an improved SPA called GSPA was proposed to make hazard assessment for biomass gasification stations. Owing to assessment indices were set based on immediate causes of accidents in the biomass gasification station, hence it was a specific hazard assessment in the study of Yan *et al*. [42]. In order to make a more comprehensive and overall hazard assessment for biomass gasification stations, the causes, *i*.*e*., hazard factors as many as possible should be considered for the confirmation of assessment indices. In retrospect, data of the grade classification are critical in the confirmation of assessment indices in traditional SPA. For example, Wang *et al*. [27] provided the data of liquefaction classification standard in their study for the evaluation of liquefaction using SPA, and the provided data were obtained from the published literature. Similarly, the corresponding grading criterion was also provided and confirmed based on the published literature in the study of BPSPA-FM for water resources [26]. Yan *et al*. [42] utilized the national standard to achieve the classification of hazard grades in the hazard assessment for biomass gasification stations using GSPA. However, for the hazard assessment of biomass gasification stations, the corresponding classification of hazard grades of some assessment indices can't be achieved due to the national standard and existing literature are lack of related data for the classification of hazard grades. As a result, the traditional SPA needs to be improved so that the hazard assessment for biomass gasification stations can still be made when the classification of hazard grades is limited.

Therefore in this study, a modified SPA called extended set pair analysis (ESPA) is proposed to make hazard assessment for biomass gasification stations. Formulas to calculate the connection degree (CD) of each sample are resolvable without any information of hazard grades classification. Then the ideal sample is employed to make comparison of the assessed samples, and the connection degree core (CDC), diversity degree (DD), and similarity degree (SD) are introduced and defined to deal with the CD so that the CD of the assessed sample can be converted into the Euclidean distance (ED). Thereby ranking the hazard of each biomass gasification station based on the calculated value of ED. After that, six biomass gasification stations in Northeast China are conducted to make hazard assessment using ESPA in the sorted hazard ranking. In contrast, six biomass gasification stations are made hazard assessment by GSPA as well [42], and the availability and validity of ESPA for the hazard assessment are proved by comparison results. For a given evaluation model, the sensitivity analysis is an effective method to observe the variation of outputs when some inputs are varied, and the reasonability of the evaluation model will be verified based on the variation trend of outputs [18,43,44]. Therefore, the sensitivity analysis is introduced to check the consistency of hazard assessment results obtained by ESPA and GSPA so that the reasonability of ESPA can be justified.

## 2. Methodology

### 2.1. Hazard assessment by SPA

The focus of the SPA is to integrate certainty and uncertainty in a given system. In the SPA, identity, discrepancy, and contradistinction are used to describe objects and their relations to each other [36]. Assuming the sets given are *A* and *B*, and the set pair *H* = (*A*, *B*) is made up of *A* and *B*, then the set pair *H* is demonstrated by its characteristics and the amount of characteristics is represented by *N*. Among these characteristics, the amounts of identity characteristics, discrepancy characteristics, and contradistinction characteristics are *S*, *F*, and *P*, respectively. Meanwhile, the values of *S*, *F*, *P*, and *N* meet the condition *N* = *S*+*F*+*P*. Thus, the values for *S*/*N*, *F*/*N* and *P*/*N* are called the identity, discrepancy, and contradistinction degrees, respectively. For convenience, let *a* = *S*/*N*, *b* = *F*/*N*, and c = *P*/*N*. Obviously, the values of *a*, *b*, and *c* satisfy the condition *a*+*b*+*c* = 1. Then the CD is defined to describe the relationship of these characteristics, and it is calculated by Eq 1,

$$\mu =\frac{S}{N}+\frac{F}{N}i+\frac{P}{N}j=a+bi+cj$$ (1)

where *μ* denotes CD, *a* denotes identity degree, *b* denotes discrepancy degree, *c* denotes contradistinction degree, *i* denotes the uncertainty coefficient of discrepancy and the range of its value is [–1,1], *j* denotes contradictory coefficient and its value is defined as -1 [35].

Generally speaking, many assessment indices are presented in the hazard assessment using SPA, the impact of each index have different weight. Therefore, the total CD considering weight is calculated by Eq 2,

$$\mu_{s}=\sum_{k=1}^{m}\mu_{k}\omega_{k}$$ (2)

where *μ*_*s*_ denotes the total CD, *ω*_*k*_ denotes the weight of index *k*, and *μ*_*k*_ denotes the CD of index *k*, *m* denotes the number of indices.

Finally, the hazard grade is confirmed by the maximal CD principle. For example, assuming the CD of sample *l* is *μ*_*l*_ *= a+bi+cj*, corresponding hazard grades are *a*-safety, *b*-middle hazard, and *c*-hazard. If the value of *c* is the maximal, then the hazard grade of sample *l* can be confirmed as 'hazard'.

### 2.2. Hazard assessment by ESPA

**Fig 1:**
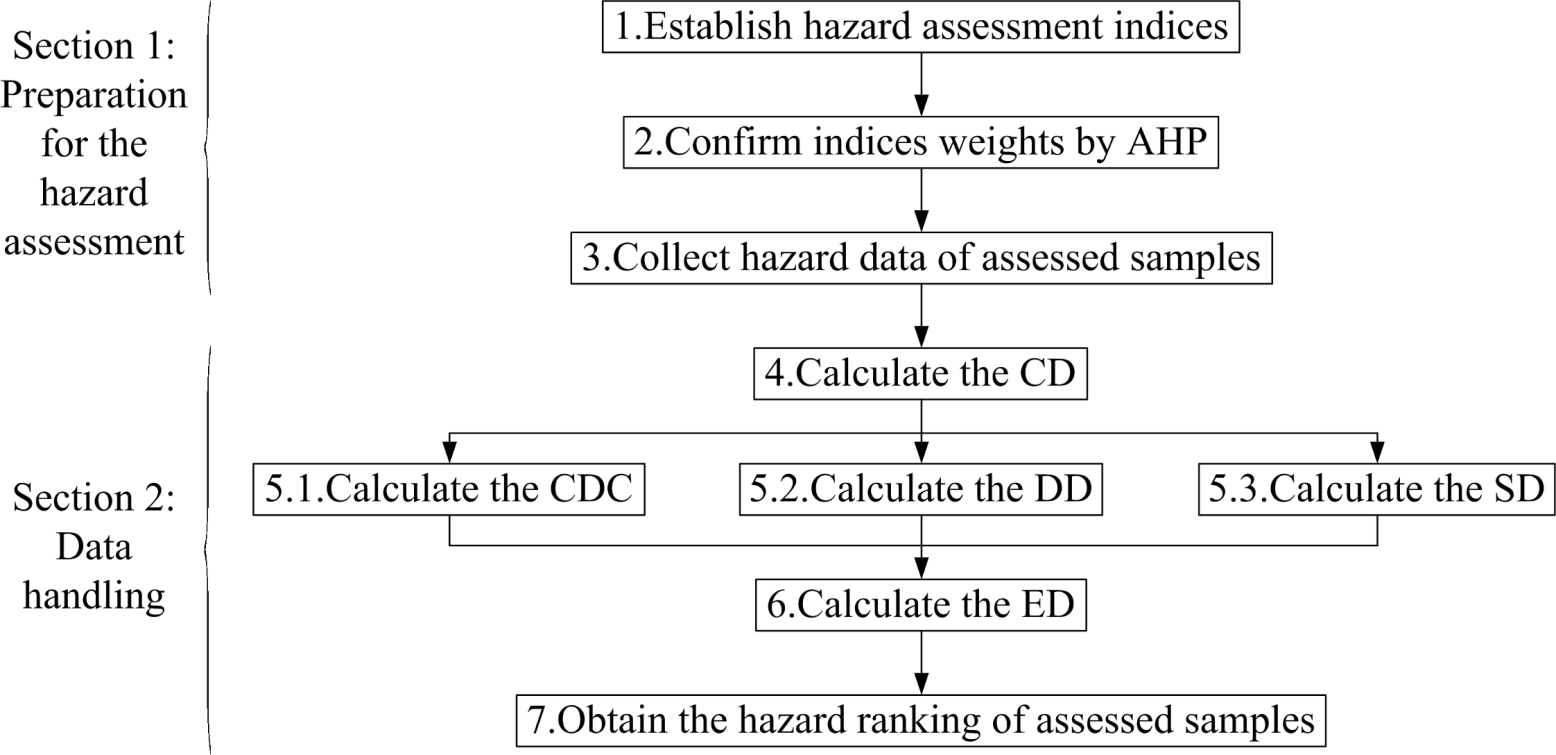
The flowchart of the hazard assessment by ESPA.

The core of SPA is the calculation of the CD. As it can be concluded from the previous work related to SPA [21,36,42], hazard grades and their corresponding thresholds are necessary for the calculation of the CD. But in some cases, the samples can't be made hazard assessment by SPA due to hazard grades and their corresponding thresholds are unknown. In this study, an improved approach called ESPA is proposed, and the proposed ESPA can make hazard assessment for samples when hazard grades and their corresponding thresholds are unknown, and the hazard of each sample can be compared and ranked based on the ESPA. A flowchart is provided in Fig 1. As it is shown in Fig 1, the procedure of the hazard assessment by ESPA include two sections and seven steps, and hazard assessment results are shown as hazard rankings.

#### 2.2.1. Confirmation of indices weights by AHP

Owing to the AHP had been widely used in the confirmation of indices weights during the assessment by SPA [28,29,35,42], a brief introduction for the confirmation methodology of indices weights by AHP is made in this study. The overall objective, middle factors and criteria should be confirmed at first with respect to this methodology. Herein, middle factors and criteria are set as potential accidents and hazard assessment indices, respectively. Thereby setting the overall objective as the hazard assessment for the assessed object. After that, pair-wise comparisons are introduced to make judgment for assessment indices. Subsequently, judgment matrices are obtained based on the 1/9-9 scale [29]. In other words, assuming two criteria *θ*_1_ and *θ*_2_ are made comparison, if the two criteria are equally important for a middle factor, then values reflected in the judgment matrix will be $A_{\theta_{1},\theta_{2}}$ = $A_{\theta_{2},\theta_{1}}$ = 1. When the criterion *θ*_1_ is more important than *θ*_2_ for a middle factor, and the important degree is the highest, then values reflected in the judgment matrix will be $A_{\theta_{1},\theta_{2}}$ *=* 9 and $A_{\theta_{2},\theta_{1}}$ = 1/9. At last, weights are calculated by the calculation methodology of AHP [45]. It should be noticed that value of the consistency ratio (CR) for the judgment matrix must be less than 0.1 based on the principle of AHP [45]. If the value of the CR isn't less than 0.1, then the judgment must be adjusted until the value of the CR is less than 0.1.

#### 2.2.2. Calculation of CD by ESPA

If the confirmation of hazard grades and their corresponding thresholds is limited, then the CD cannot be calculated by SPA. Nevertheless, the CD can still be calculated using ESPA only based on the data of the assessed samples. Assuming several samples are made hazard assessment using ESPA. For the index *k*, if the greater value of the data means a lower level of the hazard, then the smaller value of the data means a higher level of the hazard. For the data of samples in the index *k*, let the greatest and smallest value of the data be the upper threshold *u*_*k*_ and lower threshold *v*_*k*_, respectively. Assuming an arbitrary value *x*_*kl*_ belongs to [*v*_*k*_, *u*_*k*_], Eq 3 or Eq 4 both can be defined to compute the closeness degree of *x*_*kl*_ to *u*_*k*_ [46].

$$\varphi_{u_{k}1}=\frac{x_{kl}-v_{k}}{u_{k}-v_{k}}$$ (3)

$$\varphi_{u_{k}2}=\frac{x_{kl}}{u_{k}+v_{k}}$$ (4)

where *φ* denotes the closeness degree.

Owing to the sum of the closeness degree of *x*_*kl*_ to *u*_*k*_ and *v*_*k*_ is 1, therefore, the corresponding closeness degree of *x*_*kl*_ to *v*_*k*_ can be defined as Eq 5 or Eq 6 [46].

$$\varphi_{v_{k}1}=1-\varphi_{u_{k}1}=1-\frac{x_{kl}-v_{k}}{u_{k}-v_{k}}=\frac{u_{k}-x_{kl}}{u_{k}-v_{k}}$$ (5)

$$\varphi_{v_{k}2}=1-\varphi_{u_{k}2}=1-\frac{x_{kl}}{u_{k}+v_{k}}=\frac{u_{k}+v_{k}-x_{kl}}{u_{k}+v_{k}}$$ (6)

Then, let the product of Eq 3 and Eq 4 be the closeness degree of *x*_*kl*_ to *u*_*k*_ (Eq 7).

$$\varphi_{u_{k}}=\frac{x_{kl}-v_{k}}{u_{k}-v_{k}}\cdot \frac{x_{kl}}{u_{k}+v_{k}}=\frac{x_{kl}(x_{kl}-v_{k})}{\left(u_{k}-v_{k})(u_{k}+v_{k}\right)}$$ (7)

Let the product of Eq 5 and Eq 6 be the closeness degree of *x*_*kl*_ to *v*_*k*_ (Eq 8).

$$\varphi_{v_{k}}=\frac{u_{k}-x_{kl}}{u_{k}-v_{k}}\cdot \frac{u_{k}+v_{k}-x_{kl}}{u_{k}+v_{k}}=\frac{\left(u_{k}-x_{kl})(u_{k}+v_{k}-x_{kl}\right)}{\left(u_{k}-v_{k})(u_{k}+v_{k}\right)}$$ (8)

After that, the two closeness degrees are combined and denoted as a function (Eq 9),

$$f(x_{kl})=\frac{x_{kl}(x_{kl}-v_{k})+(u_{k}-x_{kl})(u_{k}+v_{k}-x_{kl})}{\left(u_{k}-v_{k})(u_{k}+v_{k}\right)}$$ (9)

Then the first-order derivative and the second-order derivative of Eq 9 are calculated (Eq 10 and Eq 11),

$$\frac{\partial f(x_{kl})}{\partial x_{kl}}=\frac{2(2x_{kl}-u_{k}-v_{k})}{\left(u_{k}-v_{k})(u_{k}+v_{k}\right)}$$ (10)

$$\frac{\partial^{2}f(x_{kl})}{\partial x_{kl}^{2}}=4$$ (11)

Obviously, it can be concluded that Eq 9 will get the maximum value when the value of *x*_*kl*_ is *u*_*k*_ or *v*_*k*_, then the maximum value of Eq 9 is shown by Eq 12.

$$f{\left(x_{kl}\right)}_{\max}=f(u_{k})=f(v_{k})=\frac{u_{k}}{u_{k}+v_{k}}$$ (12)

The closeness degree of *x*_*kl*_ to *u*_*k*_ is set to be the identity degree, meanwhile, the closeness degree of *x*_*kl*_ to *v*_*k*_ is set to be the contradistinction degree. As it has been mentioned in section 2.1., values of the identity degree, discrepancy degree, and contradistinction degree must belong to [0,1]. Accordingly, the maximum value obtained by Eq 12 is set as the quotient to make normalization of the closeness degrees for *x*_*kl*_ to *u*_*k*_ and *x*_*kl*_ to *v*_*k*_ so that values of them are in [0,1] (Eq 13 and Eq 14).

$$a=\frac{x_{kl}(x_{kl}-v_{k})}{\left(u_{k}-v_{k})(u_{k}+v_{k}\right)}/\frac{u_{k}}{u_{k}+v_{k}}=\frac{x_{kl}(x_{kl}-v_{k})}{u_{k}(u_{k}-v_{k})}$$ (13)

$$c=\frac{\left(u_{k}-x_{kl})(u_{k}+v_{k}-x_{kl}\right)}{\left(u_{k}-v_{k})(u_{k}+v_{k}\right)}/\frac{u_{k}}{u_{k}+v_{k}}=\frac{\left(u_{k}-x_{kl})(u_{k}+v_{k}-x_{kl}\right)}{u_{k}(u_{k}-v_{k})}$$ (14)

As previously mentioned in section 2.1., the discrepancy degree can be worked out based on the condition *a*+*b*+*c* = 1 (Eq 15).

$$\begin{array}{l} b=1-a-c\\ \;=1-\frac{x_{kl}(x_{kl}-v_{k})}{u_{k}(u_{k}-v_{k})}-\frac{\left(u_{k}-x_{kl})(u_{k}+v_{k}-x_{kl}\right)}{u_{k}(u_{k}-v_{k})}\\ \;=\frac{2(u_{k}-x_{kl})(x_{kl}-v_{k})}{u_{k}(u_{k}-v_{k})} \end{array}$$ (15)

Finally, the CD of the sample *l* with respect to the index *k* can be calculated by Eq 16.

$$\mu_{kl}=\frac{x_{kl}(x_{kl}-v_{k})}{u_{k}(u_{k}-v_{k})}+\frac{2(u_{k}-x_{kl})(x_{kl}-v_{k})}{u_{k}(u_{k}-v_{k})}i+\frac{\left(u_{k}-x_{kl})(u_{k}+v_{k}-x_{kl}\right)}{u_{k}(u_{k}-v_{k})}j$$ (16)

Conversely, if the greater values of data mean a higher level of the hazard, and the smaller values of the data mean a lower level of the hazard, then the CD of the sample *l* with respect to the index *k* can be calculated by Eq 17.

$$\mu_{kl}=\frac{\left(u_{k}-x_{kl})(u_{k}+v_{k}-x_{kl}\right)}{u_{k}(u_{k}-v_{k})}+\frac{2(u_{k}-x_{kl})(x_{kl}-v_{k})}{u_{k}(u_{k}-v_{k})}i+\frac{x_{kl}(x_{kl}-v_{k})}{u_{k}(u_{k}-v_{k})}j$$ (17)

#### 2.2.3. Ranking the hazard of each sample

After the CD of each sample is confirmed, the hazard of each sample can be ranked by comparing the value of the CD. In regard to a confirmed CD *μ*, the CDC is introduced to analyze a certain characteristic of the set pair *H* = (*A*, *B*), then the identity and contradistinction are made comparison by it. The CDC is important to reflect the characteristic of SPA, and Eq 18 is defined to calculate it.

C(μ)=a−c (18)

where *C*(*μ*) denotes the CDC for the CD of *μ*.

In order to rank the hazard of each sample, the CD of each sample needs to be compared. Hence the DD is proposed to describe the diversity of a couple of samples. Assuming two hazard assessment samples *x* and *y*, CDs of them are *μ*_*x*_ = *a*_*x*_+*b*_*x*_*i*+*c*_*x*_*j* and *μ*_*y*_ = *a*_*y*_+*b*_*y*_*i*+*c*_*y*_*j*, respectively. Then DDs of the CDC, identity, discrepancy, and contradistinction are calculated by Eq 19 through 22.

$$D_{CDC}(\mu_{x},\mu_{y})=\left|C(\mu_{x})-C(\mu_{y})\right|$$ (19)

$$D_{a}(\mu_{x},\mu_{y})=\left|a_{x}-a_{y}\right|$$ (20)

$$D_{b}(\mu_{x},\mu_{y})=\left|b_{x}-b_{y}\right|$$ (21)

$$D_{c}(\mu_{x},\mu_{y})=\left|c_{x}-c_{y}\right|$$ (22)

where *D*_*CDC*_(*μ*_*x*_, *μ*_*y*_), *D*_*a*_(*μ*_*x*_, *μ*_*y*_), *D*_*b*_(*μ*_*x*_, *μ*_*y*_), and *D*_*c*_(*μ*_*x*_, *μ*_*y*_) denote the DD of CDC, identity, discrepancy, and contradistinction for *μ*_*x*_ and *μ*_*y*_, respectively.

After that, the SD is proposed to reflect the similarity of a couple of samples. Eq 23 is used to calculate the SD with respect to *μ*_*x*_ and *μ*_*y*_, and it can be seen that the range of SD is [0, 1]. When the value of SD is closer to 1, it indicates that the sample *x* and sample *y* are more similar. On the contrary, the sample *x* and sample *y* are more different.

$$S(\mu_{x},\mu_{y})=1-\frac{D_{CDC}(\mu_{x},\mu_{y})+D_{a}(\mu_{x},\mu_{y})+D_{b}(\mu_{x},\mu_{y})+D_{c}(\mu_{x},\mu_{y})}{4}$$ (23)

where *S*(*μ*_*x*_, *μ*_*y*_) denotes the SD of the CD for sample *x* and sample *y*.

Then the ideal sample is employed to make a further processing for the SD. The ideal sample denotes a sample which is absolute safety or absolute hazardous, and it is used to make comparison with the samples which are made hazard assessment. As the definition of SPA [23], the CDs of the absolute safety ideal sample and the absolute hazardous ideal sample are defined as Eq 24 and Eq 25, respectively.

$$\mu_{\mathrm{safety}}=1+0\cdot i+0\cdot j$$ (24)

$$\mu_{\mathrm{hazardous}}=0+0\cdot i+1\cdot j$$ (25)

where *μ*_safety_ denotes the CD of the absolute safety ideal sample, *μ*_hazardous_ denotes the CD of the absolute hazardous ideal sample.

The ED is introduced to evaluate the relevance of the assessed samples and the ideal sample afterwards. As a straight-line distance metric [47], the ED can be used to assess the relevance of fuzzy linguistic variables [48]. Moreover, it can also be used in the anomaly detection of mechanical systems and other fields [49]. Thereby using the ED to evaluate the SD of each assessed sample in this study so that the relevance of the assessed samples and the ideal sample can be confirmed. However, as multiple indices are considered in the hazard assessment using ESPA, and the weight of each index is different in general, therefore, the index weight is considered in the calculation of the ED (Eq 26).

$$d(\mu_{l},u^{*})={\left\{{\sum_{k=1}^{m}\left[\omega_{k}\left(1-S(\mu_{kl},u^{*})\right)\right]}^{2}\right\}}^{\frac{1}{2}}$$ (26)

where *d*(*μ*_*l*_, μ^*^) denotes the ED of the CD between the assessed sample *l* and the ideal sample, *μ*_*kl*_ denotes the CD of the assessed sample *l* in index *k*, μ^*^ denotes the CD of the ideal sample.

Obviously, if the ideal sample is absolute safety, then the greater value of the ED means the higher hazard of the sample *l*, or the smaller value of the ED means the lower hazard of the sample *l*. On the contrary, if the ideal sample is absolute hazardous, a contrary conclusion can be got. Finally, the hazard ranking can be achieved based on the value of ED.

## 3. Case study

### 3.1. Confirmation of hazard assessment indices and calculation of indices weights

**Fig 2:**
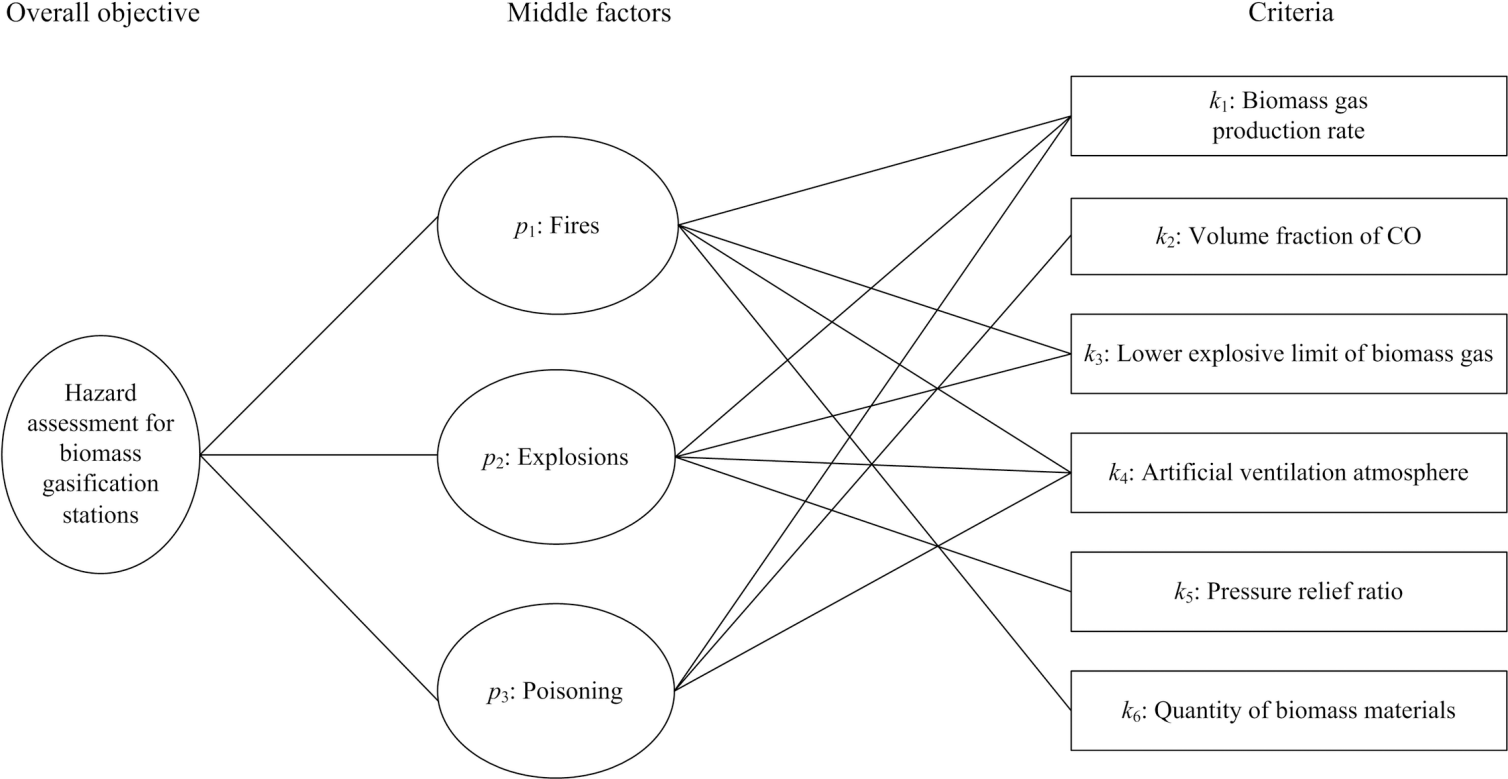
Hierarchy construction model.

In this study, no specific permissions were required for the locations introduced. Because these locations are public area and our activities were permitted by Shenyang Municipality. We can ensure that the field studies did not involve endangered or protected species. Then the hazard assessment was made for biomass gasification stations using ESPA. In order to make preparation for the hazard assessment by ESPA, hazard assessment indices and indices weights should be confirmed firstly. In the biomass gasification system, biomass materials and generated biomass gas result in various hazard factors. Owing to biomass materials are burned with insufficient oxygen to produce the biomass gas, therefore, the generated biomass gas contains flammable gases hydrogen (H_2_), carbon monoxide (CO), and methane (CH_4_) with CO having a high poisonousness as well [10]. As a result, potential accidents in biomass gasification stations are mainly involved in fires, explosions, and poisoning, thereby setting them to be middle factors *p*_1_, *p*_2_ and *p*_3_. In order to verify the availability, validity and reasonability of the hazard assessment results obtained by ESPA, methods proposed in Yan's work [42] are employed to make comparison in the following contents. Hence the confirmation of criteria is referred to Yan's work [42]. That is to say, criteria, i.e., hazard assessment indices are set to be biomass gas production rate (*k*_1_), volume fraction of CO (*k*_2_), lower explosive limit of biomass gas (*k*_3_), artificial ventilation atmosphere (*k*_4_), pressure relief ratio (*k*_5_), and quantity of biomass materials (*k*_6_). It should be stated that above six indices are immediate causes for the fire, explosion and poisoning of biomass gasification stations. Hence they are set to be criteria of AHP. The hierarchy construction (Fig 2) and the calculation of indices weights are also referred to Yan's work (Eq 27 through 30; Table 1) [42].

**Table 1:** Indices weights.

Index	Weight
Biomass gas production rate (*k*_1_)	0.1586
Volume fraction of CO (*k*_2_)	0.0293
Lower explosive limit of biomass gas (*k*_3_)	0.3594
Artificial ventilation atmosphere (*k*_4_)	0.3273
Pressure relief ratio (*k*_5_)	0.0985
Quantity of biomass materials (*k*_6_)	0.0269

$$M_{1}=\left[\begin{array}{ccc} A_{p_{1},p_{1}} & A_{p_{1},p_{2}} & A_{p_{1},p_{3}}\\ A_{p_{2},p_{1}} & A_{p_{2},p_{2}} & A_{p_{2},p_{3}}\\ A_{p_{3},p_{1}} & A_{p_{3},p_{2}} & A_{p_{3},p_{3}} \end{array}\right]=\left[\begin{array}{ccc} 1 & 1/3 & 1\\ 3 & 1 & 2\\ 1 & 1/2 & 1 \end{array}\right]$$ (27)

$$M_{2}=\left[\begin{array}{cccc} A_{k_{1},k_{1}} & A_{k_{1},k_{3}} & A_{k_{1},k_{4}} & A_{k_{1},k_{6}}\\ A_{k_{3},k_{1}} & A_{k_{3},k_{3}} & A_{k_{3},k_{4}} & A_{k_{3},k_{6}}\\ A_{k_{4},k_{1}} & A_{k_{4},k_{3}} & A_{k_{4},k_{4}} & A_{k_{4},k_{6}}\\ A_{k_{6},k_{1}} & A_{k_{6},k_{3}} & A_{k_{6},k_{4}} & A_{k_{6},k_{6}} \end{array}\right]=\left[\begin{array}{cccc} 1 & 1/3 & 1/2 & 1\\ 3 & 1 & 2 & 4\\ 2 & 1/2 & 1 & 2\\ 1 & 1/4 & 1/2 & 1 \end{array}\right]$$ (28)

$$M_{3}=\left[\begin{array}{cccc} A_{k_{1},k_{1}} & A_{k_{1},k_{3}} & A_{k_{1},k_{4}} & A_{k_{1},k_{5}}\\ A_{k_{3},k_{1}} & A_{k_{3},k_{3}} & A_{k_{3},k_{4}} & A_{k_{3},k_{5}}\\ A_{k_{4},k_{1}} & A_{k_{4},k_{3}} & A_{k_{4},k_{4}} & A_{k_{4},k_{5}}\\ A_{k_{5},k_{1}} & A_{k_{5},k_{3}} & A_{k_{5},k_{4}} & A_{k_{5},k_{5}} \end{array}\right]=\left[\begin{array}{cccc} 1 & 1/4 & 1/2 & 1/3\\ 4 & 1 & 2 & 3\\ 2 & 1/2 & 1 & 2\\ 3 & 1/3 & 1/2 & 1 \end{array}\right]$$ (29)

$$M_{4}=\left[\begin{array}{ccc} A_{k_{1},k_{1}} & A_{k_{1},k_{2}} & A_{k_{1},k_{4}}\\ A_{k_{2},k_{1}} & A_{k_{2},k_{2}} & A_{k_{2},k_{4}}\\ A_{k_{4},k_{1}} & A_{k_{4},k_{2}} & A_{k_{4},k_{4}} \end{array}\right]=\left[\begin{array}{ccc} 1 & 3 & 1/2\\ 1/3 & 1 & 1/4\\ 2 & 4 & 1 \end{array}\right]$$ (30)

### 3.2. Data collection

As previously mentioned, six assessment indices are introduced to make hazard assessment for biomass gasification stations using ESPA. The data collected from the biomass gasification station are used to make ESPA. Index *k*_1_ denotes the volume of produced biomass gas per hour (m^3^/h), and data of index *k*_1_ can be obtained from the production status of the biomass gasification station. Index *k*_2_ and *k*_3_ denote the volume fraction of CO in the produced biomass gas (%) and the lower explosive limit of the produced biomass gas (%), respectively. Data of them can be obtained based on the measurement of the produced biomass gas. Index *k*_4_ denotes the artificial ventilation ability of the biomass gasification station, it is reflected by air change rate (times/h). Data of index *k*_4_ can be confirmed by the artificial ventilation property of the biomass gasification station. Index *k*_5_ denotes the pressure relief ability of the biomass gasification station when fires and explosions occur. Index *k*_6_ denotes the quantity of biomass materials stored in the storage room of the biomass gasification station. All in all, data of indices *k*_5_ and *k*_6_ are all involved in the construction size of the biomass gasification station. Data of index *k*_5_ are obtained by the calculation of Eq 31 [50]. Data of index *k*_6_ are confirmed by the volume of the storage room (m^3^), in general, the value of *k*_6_ is set as one third of the volume of the storage room.

$$C=A/10V^{2/3}$$ (31)

where *C* denotes the pressure relief ratio (m^2^/m^3^), *A* denotes the area of pressure relief (m^2^), where the value of *A* is equal to the area of windows and doors in the biomass gasification station [50], and *V* is the volume of the biomass gasification station (m^3^).

Obviously, for the index *k*_3_, *k*_4_, and *k*_5_, the greater value of the data means a lower level of hazard. On the other hand, for the index *k*_1_, *k*_2_, and *k*_6_, the greater value of the data means a higher level of hazard.

In this study, six samples (*l*_1_, *l*_2_, *l*_3_, *l*_4_, *l*_5_, *l*_6_), *i*.*e*., six biomass gasification stations in Northeast China were introduced to make hazard assessment using ESPA. Above six biomass gasification stations are Huangtukan station (122.767°E, 41.718°N), Yanjia station (123.750°E, 41.996°N), Shengli station (123.343°E, 41.522°N), Wangpingfang station (123.437°E, 42.916°N), Xinli station (123.684°E, 42.582°N), and Dazhang station (122.546°E, 41.282°N). The construction size of each biomass gasification station is shown in Table 2, and index data of *k*_5_ and *k*_6_ are then calculated (Eq 31). Index data of each biomass gasification station are listed in Table 3.

**Table 2:** Construction size of six biomass gasification stations.

Construction size	*l*_1_	*l*_2_	*l*_3_	*l*_4_	*l*_5_	*l*_6_
Area of windows and doors (m^2^)	17.28	49.66	35.60	34.30	43.52	44.30
Volume of the biomass gasification station (m^3^)	142.53	328.08	240.60	163.98	364.22	506.39
Volume of the storage room (m^3^)	42.99	87.58	57.38	42.65	98.42	83.67

**Table 3:** Index data of six biomass gasification stations.

Indices	*l*_1_	*l*_2_	*l*_3_	*l*_4_	*l*_5_	*l*_6_
*k*_1_ (m^3^/h)	300	700	400	200	450	600
*k*_2_ (%)	21.46	19.69	21.69	14.91	17.41	21.73
*k*_3_ (%)	20.74	23.05	23.45	25.57	29.68	16.43
*k*_4_ (times/h)	10	6	6	11	8	8
*k*_5_ (m^2^/m^3^)	0.0633	0.1044	0.0920	0.1145	0.0853	0.0697
*k*_6_ (m^3^)	14.33	29.19	19.13	14.22	32.81	27.89

## 4. Results and discussion

### 4.1. Hazard assessment results by ESPA

It should be noted at first that the classification of hazard grades with respect to the above assessment indices are achieved according to Yan's work [42]. In order to make comparison, assuming hazard grades and their corresponding thresholds are unknown, and the hazard of each sample only can be confirmed by the collected data. Then the CD of each sample in index *k*_3_, *k*_4_, and *k*_5_ is calculated by Eq 16, meanwhile, the CD of each sample in index *k*_1_, *k*_2_, and *k*_6_ is calculated by Eq 17. Calculation results are listed in Table 4.

**Table 4:** CD of each sample in each index.

Indices	CD of each biomass gasification station	
*k*_1_	*l*_1_	*l*_2_
	*μ* = 0.6857+0.2286*i*+0.0857*j*	*μ* = 0.0000+0.0000*i*+1.0000*j*
	*l*_3_	*l*_4_
	*μ* = 0.4286+0.3429*i*+0.2286*j*	*μ* = 1.0000+0.0000*i*+0.0000*j*
	*l*_5_	*l*_6_
	*μ* = 0.3214+0.3571*i*+0.3214*j*	*μ* = 0.0857+0.2286*i*+0.6857*j*
*k*_2_	*l*_1_	*l*_2_
	*μ* = 0.0277+0.0239*i*+0.9485*j*	*μ* = 0.2333+0.1316*i*+0.6351*j*
	*l*_3_	*l*_4_
	*μ* = 0.0040+0.0037*i*+0.9923*j*	*μ* = 1.0000+0.0000*i*+0.0000*j*
	*l*_5_	*l*_6_
	*μ* = 0.5606+0.1458*i*+0.2937*j*	*μ* = 0.0000+0.0000*i*+1.0000*j*
*k*_3_	*l*_1_	*l*_2_
	*μ* = 0.2273+0.1960*i*+0.5767*j*	*μ* = 0.3880+0.2232*i*+0.3888*j*
	*l*_3_	*l*_4_
	*μ* = 0.4186+0.2224*i*+0.3590*j*	*μ* = 0.5943+0.1910*i*+0.2147*j*
	*l*_5_	*l*_6_
	*μ* = 1.0000+0.0000*i*+0.0000*j*	*μ* = 0.0000+0.0000*i*+1.0000*j*
*k*_4_	*l*_1_	*l*_2_
	*μ* = 0.7273+0.1455*i*+0.1273*j*	*μ* = 0.0000+0.0000*i*+1.0000*j*
	*l*_3_	*l*_4_
	*μ* = 0.0000+0.0000*i*+1.0000*j*	*μ* = 1.0000+0.0000*i*+0.0000*j*
	*l*_5_	*l*_6_
	*μ* = 0.2909+0.2182*i*+0.4909*j*	*μ* = 0.2909+0.2182*i*+0.4909*j*
*k*_5_	*l*_1_	*l*_2_
	*μ* = 0.0000+0.0000*i*+1.0000*j*	*μ* = 0.7319+0.1416*i*+0.1265*j*
	*l*_3_	*l*_4_
	*μ* = 0.4504+0.2203*i*+0.3293*j*	*μ* = 1.0000+0.0000*i*+0.0000*j*
	*l*_5_	*l*_6_
	*μ* = 0.3201+0.2192*i*+0.4607*j*	*μ* = 0.0761+0.0978*i*+0.8261*j*
*k*_6_	*l*_1_	*l*_2_
	*μ* = 0.9908+0.0067*i*+0.0026*j*	*μ* = 0.1059+0.1777*i*+0.7164*j*
	*l*_3_	*l*_4_
	*μ* = 0.6258+0.2202*i*+0.1540*j*	*μ* = 1.0000+0.0000*i*+0.0000*j*
	*l*_5_	*l*_6_
	*μ* = 0.0000+0.0000*i*+1.0000*j*	*μ* = 0.1544+0.2205*i*+0.6251*j*

After the CD is obtained, the DD of each sample is calculated by Eqs 19–22, and the SD is calculated by Eq 23 afterwards. Then the ED of each sample to the ideal sample is calculated by Eq 26. As previously mentioned, the ideal sample includes the absolute safety ideal sample and the absolute hazardous ideal sample, and EDs to the two ideal samples imply an same conclusion. Therefore, only one of the ideal samples is needed to make calculation. In this study, the absolute safety ideal sample (Eq 24) is chosen to make calculation. Then the SD of each sample with respect to each index is shown in Table 5, and the ED of each sample is listed in Table 6.

**Table 5:** SD of each sample in each index.

	*l*_1_	*l*_2_	*l*_3_	*l*_4_	*l*_5_	*l*_6_
*k*_1_	0.7429	0	0.5143	1	0.4107	0.1429
*k*_2_	0.0336	0.2662	0.0050	1	0.5970	0
*k*_3_	0.2763	0.4438	0.4742	0.6421	1	0
*k*_4_	0.7636	0	0	1	0.3455	0.3455
*k*_5_	0	0.7673	0.5055	1	0.3749	0.1005
*k*_6_	0.9924	0.1503	0.6808	1	0	0.2095

**Table 6:** ED of each sample.

	ED
*l*_1_	0.2929
*l*_2_	0.4168
*l*_3_	0.3900
*l*_4_	0.1286
*l*_5_	0.2435
*l*_6_	0.4502

Clearly enough, above results are achieved by a simple and concise calculation, what's more, the CD can be obtained even though hazard grades and its corresponding thresholds are unknown. As the greater value of the ED means the higher hazard of the sample here, thus it can be concluded that the hazard ranking of biomass gasification stations is *l*_**6**_**>***l*_**2**_**>***l*_**3**_**>***l*_**1**_**>***l*_**5**_**>***l*_**4**_.

### 4.2. Hazard assessment results by GSPA

Obviously, if the confirmation of hazard grades and their corresponding thresholds are limited, the hazard of each biomass gasification station can still be evaluated by the proposed ESPA in this study. According to Yan's study [42], the hazard of each biomass gasification station can be evaluated by GSPA when hazard grades and their corresponding thresholds are known. In order to verify the availability and validity of hazard assessment results using ESPA, GSPA is introduced to rank the hazard of each biomass gasification station. Owing to the assessment indices and their index weights employed in this study are same to that of Yan's [42], a direct processing can be made for the data with respect to biomass gasification stations by GSPA. Then the connection measure degree (CMD) and comprehensive index (CI) are calculated by GSPA (Table 7; Table 8) [42].

**Table 7:** CMD of each sample.

	*μ*_I_	*μ*_II_	*μ*_III_	*μ*_IV_	*μ*_V_	*μ*_I_	*μ*_II_	*μ*_III_	*μ*_IV_	*μ*_V_
*k*_1_	*l*_1_					*l*_2_				
	1	0	-1	-1	-1	0.3090	1	-0.3090	-1	-1
	*l*_3_					*l*_4_				
	1	0.7071	-1	-1	-1	1	-0.7071	-1	-1	-1
	*l*_5_					*l*_6_				
	1	0.9239	-1	-1	-1	0.8090	1	-0.8090	-1	-1
*k*_2_	*l*_1_					*l*_2_				
	-1	-1	-1	0.9984	1	-1	-1	-0.9811	1	0.9811
	*l*_3_					*l*_4_				
	-1	-1	-1	0.9978	1	-1	-0.9984	1	0.9984	-1
	*l*_5_					*l*_6_				
	-1	-1	0.0565	1	-0.0565	-1	-1	-1	0.9977	1
*k*_3_	*l*_1_					*l*_2_				
	-0.9731	1	0.9731	-1	-1	-0.5750	1	0.5750	-1	-1
	*l*_3_					*l*_4_				
	-0.4679	1	0.4679	-1	-1	0.1781	1	-0.1781	-1	-1
	*l*_5_					*l*_6_				
	0.9950	1	-0.9950	-1	-1	-1	-0.6228	1	0.6228	-1
*k*_4_	*l*_1_					*l*_2_				
	-0.5	1	0.5	-1	-1	-1	-1	1	1	-1
	*l*_3_					*l*_4_				
	-1	-1	1	1	-1	0.5	1	-0.5	-1	-1
	*l*_5_					*l*_6_				
	-1	0.5	1	-0.5	-1	-1	0.5	1	-0.5	-1
*k*_5_	*l*_1_					*l*_2_				
	-1	-1	-1	-0.2601	1	-1	-1	-1	0.9759	1
	*l*_3_					*l*_4_				
	-1	-1	-1	0.7604	1	-1	-1	-0.9603	1	0.9603
	*l*_5_					*l*_6_				
	-1	-1	-1	0.5653	1	-1	-1	-1	-0.0118	1
*k*_6_	*l*_1_					*l*_2_				
	≈1	1	≈-1	-1	-1	0.9999	1	-0.9999	-1	-1
	*l*_3_					*l*_4_				
	≈1	1	≈-1	-1	-1	≈1	1	≈-1	-1	-1
	*l*_5_					*l*_6_				
	0.9999	1	-0.9999	-1	-1	0.9999	1	-0.9999	-1	-1

**Table 8:** CI of each sample.

Sample	*l*_1_	*l*_2_	*l*_3_	*l*_4_	*l*_5_	*l*_6_
*CI*	2.6853	2.9550	2.8202	2.0684	2.2375	2.9575

The hazard ranking can be reflected by values of the CI because it was mentioned in Yan's study [42]. As the greater value of the CI indicates the higher hazard of the sample, therefore, the hazard ranking of each biomass gasification station is *l*_6_>*l*_2_>*l*_3_>*l*_1_>*l*_5_>*l*_4_.

In regard to the hazard assessment using ESPA, the confirmation of the hazard ranking depends on values of the ED, meanwhile, the hazard ranking is reflected by values of the CI with respect to the hazard assessment using GSPA. As it can be seen from the above calculation results, hazard assessment results, *i*.*e*., the hazard ranking of each biomass gasification station obtained by ESPA and GSPA are consistent. As GSPA is a method which can be used in the hazard assessment for biomass gasification stations [42], hence the availability and validity of ESPA can be verified. In addition, owing to hazard grades and their corresponding thresholds aren’t needed in the hazard assessment using ESPA, the scope of the application will be wider.

### 4.3. Sensitivity analysis

**Fig 3:**
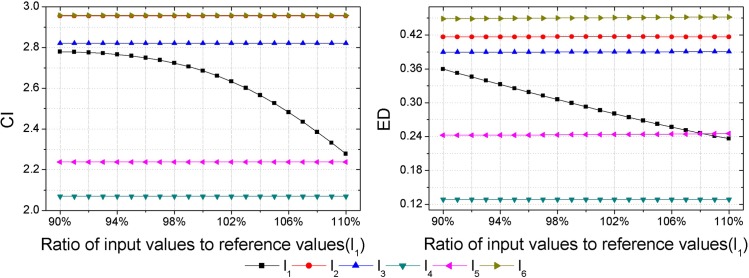
Calculation results of the CI and ED under the reference values ±10% for sample *l*_1_.

**Fig 4:**
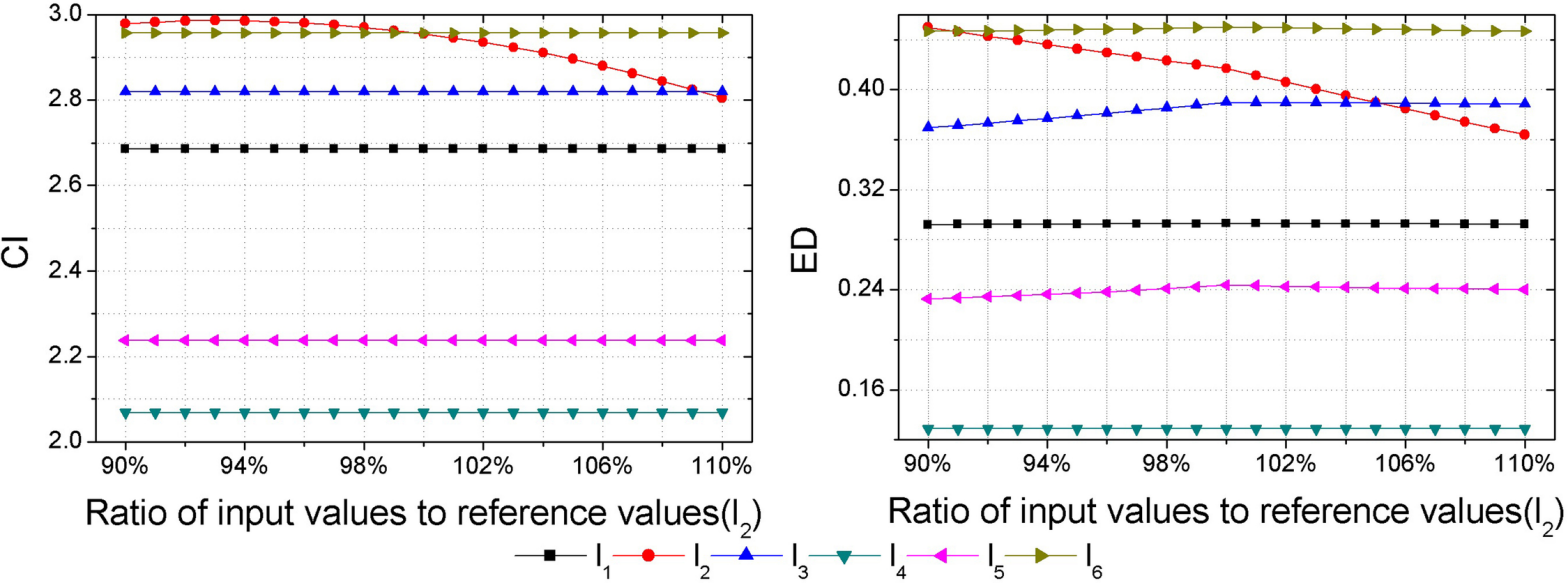
Calculation results of the CI and ED under the reference values ±10% for sample *l*_2_.

**Fig 5:**
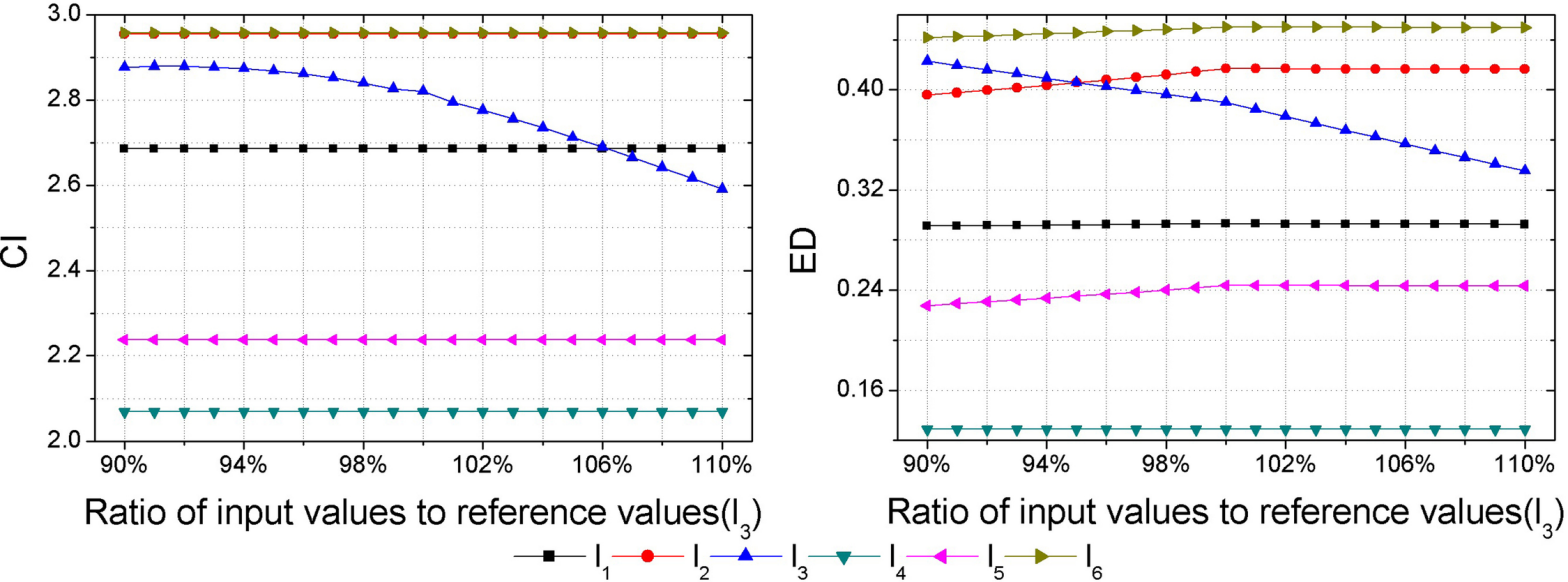
Calculation results of the CI and ED under the reference values ±10% for sample *l*_3_.

**Fig 6:**
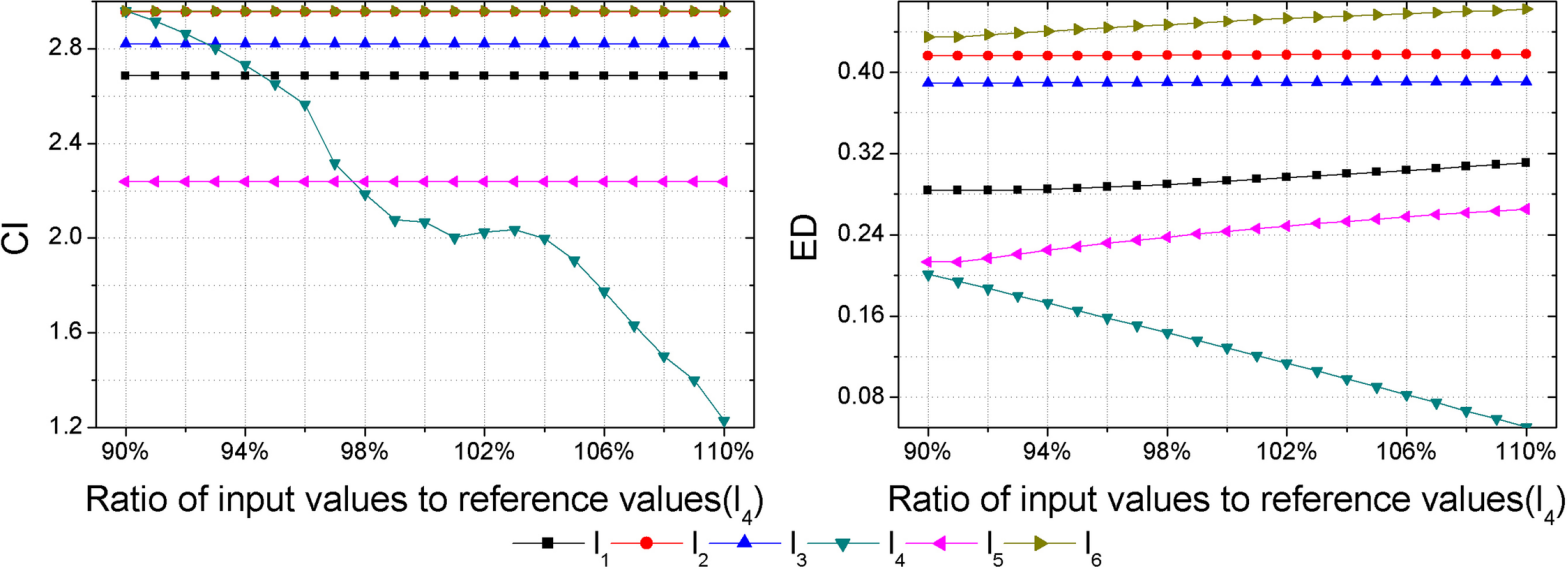
Calculation results of the CI and ED under the reference values ±10% for sample *l*_4_.

**Fig 7:**
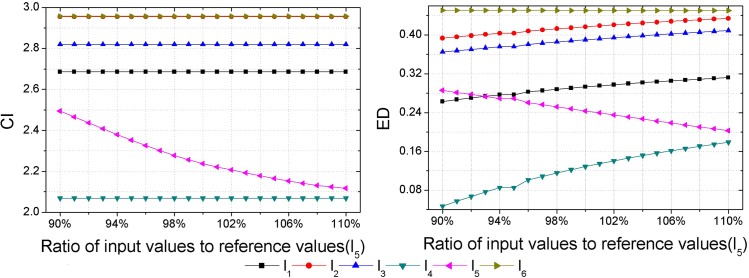
Calculation results of the CI and ED under the reference values ±10% for sample *l*_5_.

**Fig 8:**
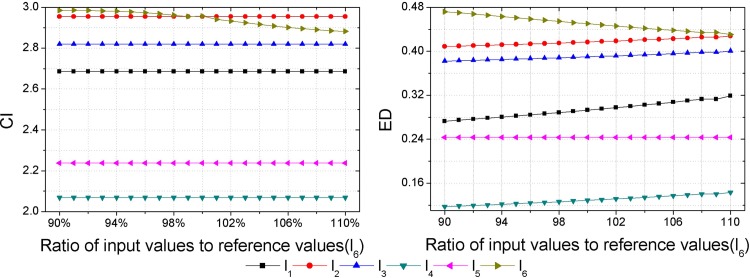
Calculation results of the CI and ED under the reference values ±10% for sample *l*_6_.

As inputs of some hazard assessment indices are uncertain, hazard assessment results will be affected by the uncertainties of them. Thus, a sensitivity analysis should be performed to check the consistency of the obtained hazard ranking. Each sample is set as the variable sample in turn, and data of the variable sample in each index are set as the variable inputs, then outputs, i.e., the CI and ED are calculated with different input values. Assuming an error of ±10% in the inputs determined [18], that is to say, the range of input values is between 90% and 110% of the reference values presented in Table 3. Herein, the interval of input values is set as 1%. Then calculations of the CI and ED with different input values are made by GSPA and ESPA, respectively (Figs 3–8).

Sensitivity analysis results show that outputs change as inputs change. In regard to the sensitivity analysis for the hazard assessment results by GSPA, only the sample which is set to be the variable input changes as inputs change. However, sensitivity analysis results of the hazard assessment results by ESPA show that all samples change as inputs change. The difference is due to the distinct computing algorithm of the CD from one another. The calculation of the CD by GSPA is based on the hazard grades and their corresponding thresholds, the change of data of a sample won't affect the other samples. On the contrary, the calculation of the CD by ESPA is based on the data of each sample so that the change of data of a sample will affect the other samples. Nevertheless, the obtained hazard rankings by GSPA and ESPA are similar. As it can be seen in Fig 3, even though values of the CI and ED for the variable sample are changed, all the relative hazard rankings of the other samples are changeless. In addition, it can be concluded by sensitivity analysis results that trends of the hazard ranking variation for an arbitrary variable sample obtained by GSPA and ESPA are coherent. In order to make a clearer observation of the hazard ranking for each variable sample, the hazard rankings of each variable sample are listed in Table 9. For variable samples *l*_1_ and *l*_5_, the hazard rankings obtained by GSPA and ESPA are almost the same. In regard to variable samples *l*_2_, *l*_3_, *l*_4_ and *l*_6_, although some hazard rankings obtained by GSPA and ESPA aren't consistent, trends of the hazard ranking variation are still coherent. For example, the hazard ranking obtained by ESPA for the variable sample *l*_4_ is always '6'. However, the trend of the hazard ranking variation shows that the smaller of the ratio of input values to reference values leads to a higher value of the ED for *l*_4_, that is to say, the hazard ranking obtained by ESPA of *l*_4_ will rise when the ratio of input values to reference values gets smaller, and this trend meets the hazard ranking variation according to GSPA.

**Table 9:** Hazard ranking variation of the variable sample.

Ratio of input values to reference values	Hazard ranking of the variable sample											
	*l*_1_		*l*_2_		*l*_3_		*l*_4_		*l*_5_		*l*_6_	
	CI	ED	CI	ED	CI	ED	CI	ED	CI	ED	CI	ED
90%	4	4	1	1	3	2	1	6	5	4	1	1
91%	4	4	1	2	3	2	3	6	5	4	1	1
92%	4	4	1	2	3	2	3	6	5	4	1	1
93%	4	4	1	2	3	2	4	6	5	5	1	1
94%	4	4	1	2	3	2	4	6	5	5	1	1
95%	4	4	1	2	3	2	5	6	5	5	1	1
96%	4	4	1	2	3	3	5	6	5	5	1	1
97%	4	4	1	2	3	3	5	6	5	5	1	1
98%	4	4	1	2	3	3	6	6	5	5	1	1
99%	4	4	1	2	3	3	6	6	5	5	1	1
100%	4	4	2	2	3	3	6	6	5	5	1	1
101%	4	4	2	2	3	3	6	6	5	5	2	1
102%	4	4	2	2	3	3	6	6	5	5	2	1
103%	4	4	2	2	3	3	6	6	5	5	2	1
104%	4	4	2	2	3	3	6	6	5	5	2	1
105%	4	4	2	2	3	3	6	6	5	5	2	1
106%	4	4	2	3	3	3	6	6	5	5	2	1
107%	4	4	2	3	4	3	6	6	5	5	2	1
108%	4	4	2	3	4	3	6	6	5	5	2	1
109%	4	5	2	3	4	3	6	6	5	5	2	1
110%	4	5	3	3	4	3	6	6	5	5	2	1

To sum up, as hazard assessment results obtained by GSPA and ESPA meet a satisfied consistency, the reasonability of ESPA can be justified based on the reasonability of GSPA [42].

### 4.4. Further work

Six assessment indices were introduced to make hazard assessment for biomass gasification stations using ESPA in this study, meanwhile, hazard grades and their corresponding thresholds of the six assessment indices were assumed to be unknown. Actually, hazard grades and their corresponding thresholds of the six assessment indices are known according to the previous study [42,50–52]. The six assessment indices introduced in this study were used to verify the availability of ESPA in the hazard assessment. However, there are many other assessment indices existed in the hazard assessment for biomass gasification stations. For most of them, hazard grades and their corresponding thresholds are unknown. In our future work, other hazard assessment indices will be added in the hazard assessment for biomass gasification stations using ESPA, these hazard assessment indices include the tar content in the produced biomass gas, human error factors, weather factors, multiple surrounding factors of the biomass gasification station and so on. Thus a more comprehensive hazard assessment will be made for biomass gasification stations.

Moreover, as it has been discussed previously, the proposed ESPA has a broad applicability so that it can be applied straightforward to other hazard assessment cases. In our future work, an attempt will been made to verify the applicability of ESPA in other fields, the hazard assessment will be made for more cases using ESPA.

## 5. Conclusion

In this paper, a novel approach called the ESPA was proposed to make hazard assessment for biomass gasification stations, which can be used to rank the hazard of each biomass gasification station. The proposed ESPA can convert hazard data of each sample into the CD when the confirmation of hazard grades and their corresponding thresholds is limited. It will overcome the restrict in the confirmation of the CD when some assessment indices are lack of data with respect to the classification of hazard grades. Hence the hazard assessment by ESPA will achieve a wider application scope. Moreover, The hazard ranking of each sample can be confirmed immediately by a simple and concise calculation of the introduced ED. By contrast with hazard assessment results of biomass gasification stations using GSPA, the availability and validity of the ESPA were verified in this study. Sensitivity analysis results showed that hazard assessment results obtained by GSPA and ESPA met a satisfied consistency, thus the reasonability of the ESPA for the hazard assessment of biomass gasification stations was justified. Characteristics indicate that the application scope of the proposed ESPA will be wide, and it may be applied in other fields involved in the hazard assessment.
